# Ultra-High Resolution Imaging Method for Distributed Small Satellite Spotlight MIMO-SAR Based on Sub-Aperture Image Fusion

**DOI:** 10.3390/s21051609

**Published:** 2021-02-25

**Authors:** Fang Zhou, Jun Yang, Lu Jia, Xingming Yang, Mengdao Xing

**Affiliations:** 1 School of Computer and Information, Hefei University of Technology, Hefei 230009, China; yangjun_anqing@163.com (J.Y.); lujia@hfut.edu.cn (L.J.); xmyang168@163.com (X.Y.); 2 Institute of Electronic Engineering, Xidian University, Xi’an 710071, China; xmd@xidian.edu.cn

**Keywords:** distributed small satellite, spotlight multiple input multiple output synthetic aperture radar (MIMO-SAR), time domain bandwidth synthesis (TBS), sub-aperture image coherent fusion

## Abstract

Small satellite synthetic aperture radar (SAR) has become a new development direction of spaceborne SAR due to its advantages of flexible launch, short development cycle, and low cost. However, there are fewer researches on distributed small satellite multiple input multiple output (MIMO) SAR. This paper proposes an ultra-high resolution imaging method for the distributed small satellite spotlight MIMO-SAR, which applies the sub-aperture division technique and the sub-aperture image coherent fusion algorithm to MIMO-SAR. After deblurring the sub-aperture signal, the large bandwidth signal is obtained by using an improved time domain bandwidth synthesis (TBS) method, and then the ultra-high resolution image is obtained by using a sub-aperture image coherent fusion algorithm. Simulation results validate the feasibility and effectiveness of the proposed approach.

## 1. Introduction

Multiple-input multiple-output synthetic aperture radar (MIMO-SAR) overcomes the contradiction between high resolution and wide swath of single-channel SAR, and is the key development direction of spaceborne SAR in the future [1,2,3,4,5]. Phased array technology is a main way to realize spaceborne MIMO-SAR operation mode [6,7], but it greatly increases the development cost of radar. Due to the advantages of flexible launch, short development cycle, and low cost, distributed small satellites have become another way to realize the working mode of spaceborne MIMO-SAR in the future [8,9,10,11]. Distributed small satellites can control the antenna beam direction, and transmit and receive signals of multiple frequency bands simultaneously to complete the configuration of spaceborne spotlight MIMO-SAR and realize high-resolution and wide swath imaging.

Spotlight mode is the main method for SAR to obtain high-resolution target image. In Spotlight mode, the Doppler bandwidth of full-aperture signal is very large. In order to avoid Doppler aliasing of echo signal, the pulse repetition rate (PRF) is generally required to be greater than this Doppler bandwidth, which will result in range ambiguity in a wide swath scenario. In order to obtain a wide swath without ambiguity, two kinds of MIMO-SAR algorithms are widely used: range ambiguity suppression algorithm and azimuth deblurring algorithm. The range ambiguity suppression algorithm is often implemented by azimuth phase coding (APC) [12,13,14]. But this kind of algorithm requires the PRF to be larger than the Doppler bandwidth of the echo. However, limited by the size and weight, the storage space of small satellites is limited [8]. If large PRF is required in ultra-high-resolution mode, the amount of data collected by radar receiver will undoubtedly increase, resulting in excessive storage pressure of satellites. Therefore, this kind of APC algorithm is not suitable for small satellites. References [15,16,17] uses the spatial degree of freedom to filter the full-aperture signal in spatial domain, so as to eliminate the azimuth ambiguity. However, when the azimuth ambiguity becomes more serious due to the improvement of resolution, it is necessary to increase the number of satellites to obtain enough spatial degrees of freedom, which will undoubtedly lead to a sharp increase in cost. In [18,19], the sub-aperture signal is deblurred, which greatly reduces the ambiguity of the processed signal, so as to ensure the sufficient degree of freedom in space. Nevertheless, such algorithms need to get full-aperture two-dimensional spectrum by frequency shift and coherent accumulation before imaging, which results in the surge of data volume and greatly increases the calculation amount of imaging processing system.

In ultra-high resolution SAR system, it is necessary to transmit ultra-wideband (UWB) signal to obtain ultra-high resolution in range yet it is difficult to generate UWB signal directly through hardware [20,21,22]. To solve this problem, one can transmit narrow-band stepped frequency signals, and then synthesize echoes in UWB signal through bandwidth synthesis technology [23,24,25,26,27,28]. Bandwidth synthesis technologies are mainly divided into two categories: time-domain bandwidth synthesis (TBS) [23] and frequency-domain bandwidth synthesis (FBS) [24,25]. The FBS method is simple to operate only when the center of the sub band and the center of the whole frequency band are separated by integer frequency points, otherwise it cannot be accurately synthesized. TBS method is characterized by higher synthesis accuracy, yet complicated process, large calculation, and low efficiency [19].

Based on the above problems, this paper proposes an ultra-high resolution imaging method for distributed small satellite spotlight MIMO-SAR, aiming to reduce the amount of data collected by satellites and the computational load of the imaging system in ultra-high resolution mode. In range, the ultra-wide band signals are obtained by transmitting stepped frequency signals and using bandwidth synthesis technology, so as to obtain ultra-high resolution. The azimuth high-resolution image is obtained by sub-aperture image fusion algorithm [29]. First, the spatial filtering technology is used to remove the azimuth ambiguity of the sub-aperture signal, and then an improved TBS method is proposed to realize the synthesis of the step frequency signal. Compared with the APC algorithms in [12,13,14], the PRF of the proposed method can be a few times of the sub-aperture Doppler bandwidth, which greatly reduces the amount of data collected by satellites. The sub-aperture processing decreases ambiguity of the processed signal, which ensures the sufficiency of the spatial degree of freedom. The improved TBS method simplifies the operation process of traditional TBS. After the two-dimensional spectrum of sub-aperture is reconstructed, the proposed method uses the sub-aperture image fusion algorithm to directly image the reconstructed sub-aperture signal, so as to obtain multiple ultra-high-resolution in range and low-resolution in azimuth images. Then these sub-aperture images are fused coherently to obtain azimuth high-resolution image corresponding to the full aperture. Compared with the imaging processing of full-aperture signal in [18,19], the imaging processing of sub-aperture signal significantly reduces the amount of data needed to be processed by the imaging system, and reduces the computational load of the imaging system.

## 2. Working Mode and Signal Model of Distributed Small Satellite Spotlight MIMO-SAR

**Figure 1:**
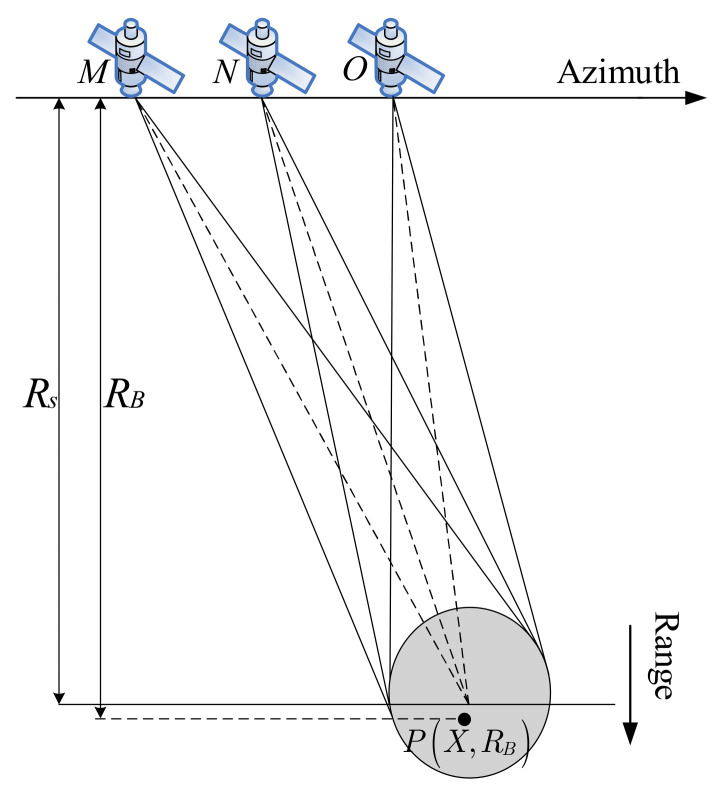
Geometric model of three transmitters and three receivers on satellite.

As shown in Figure 1, multiple satellites are linearly distributed in azimuth (without loss of generality, three satellites are taken as examples in the figure). The initial coordinates of satellites *M*, *N,* and *O* are $\left(x_{m},0\right)$, $\left(x_{n},0\right)$, and $\left(x_{o},0\right)$ respectively, and the shortest slant range of the scene center is $R_{s}$. The coordinate of a point in the scene is $P\left(X,R_{B}\right)$.

When satellite *M* transmits signal and satellite *N* receives signal, the round-trip slant range from point target P to radar transceiver is

(1) $$R_{m,n}\left(t_{a}\right)=\sqrt{R_{B}{}^{2}+{\left(X-x_{m}-vt_{a}\right)}^{2}}+\sqrt{R_{B}{}^{2}+{\left(X-x_{n}-vt_{a}\right)}^{2}}$$

where $t_{a}$ is the slow time, v is the satellite speed, m,n=1,2,⋯,Q, Q is the number of satellites. After the deviation of slant range is compensated [30], the bistatic mode of *M* and *N* can be regarded as the monostatic mode of the equivalent phase center. The equivalent phase center is located in the center of *M* and *N*, and its coordinate is $x_{m,n}=\left(x_{m}+x_{n}\right)/2$. The round-trip slant range $R_{m,n}$ is equivalent to

(2) $$R_{m,n}\left(t_{a}\right)=2\sqrt{R_{B}{}^{2}+{\left(X-x_{m,n}-vt_{a}\right)}^{2}}$$

Therefore, the signal transmitted by satellite *M* and received by satellite *N* can be expressed as

(3) $$S_{m,n}\left(\hat{t},t_{a}\right)=a_{r}\left(\hat{t}\right)\exp\left(j\pi \gamma {\left(\hat{t}-\frac{2R_{m,n}\left(t_{a}\right)}{c}\right)}^{2}\right)a_{a}\left(t_{a}\right)\exp\left(-j4\pi f_{c}\left(m\right)\frac{R_{m,n}\left(t_{a}\right)}{c}\right)$$

where γ is the chirp rate, $\hat{t}$ is the fast time, $a_{r}\left(\hat{t}\right)$ and $a_{a}\left(t_{a}\right)$ are the range window functions and azimuth window function, respectively, and $f_{c}\left(m\right)$ is the carrier frequency of the signal transmitted by satellite *M*, satisfying the equation: $f_{c}\left(m\right)=f_{c}+\left(k-1/2-m/2\right)B$. $f_{c}$ is the carrier frequency of the satellite located in the center of the satellite linear array, and B is the bandwidth of the sub-band signal. The frequency bands of sub-band signals do not overlap.

Assuming that the full aperture is divided into *K* sub apertures, the azimuth time range of the *k*th (k=1,2,⋯,K) sub-aperture is

(4) $$t_{a}\in \left[-\frac{T_{a}}{2K}:\frac{T_{a}}{2K}\right]+t_{k}k=1,2,\cdots ,K$$

where $T_{a}$ is the whole synthetic aperture time and $t_{k}$ is the central of azimuth time corresponding to the *k*th sub-aperture data. $\left[-T_{a}/2K:T_{a}/2K\right]$ is marked as $t_{sub}$, and $t_{a}$ is replaced in Equation (3) with $t_{sub}$. Then the *k*th sub-aperture signal transmitted by satellite *M* and received by satellite *N* can be expressed as

(5) $$S_{m,n}\left(\hat{t},t_{a},t_{k}\right)=a_{r}(\hat{t})\exp\left(j\pi \gamma {\left(\hat{t}-\frac{2R_{m,n}\left(t_{sub}+t_{k}\right)}{c}\right)}^{2}\right)a_{a}(t_{sub})\exp\left(-j4\pi f_{c}\left(m\right)\frac{R_{m,n}\left(t_{sub}+t_{k}\right)}{c}\right)$$

## 3. Analysis of Doppler Characteristics

**Figure 2:**
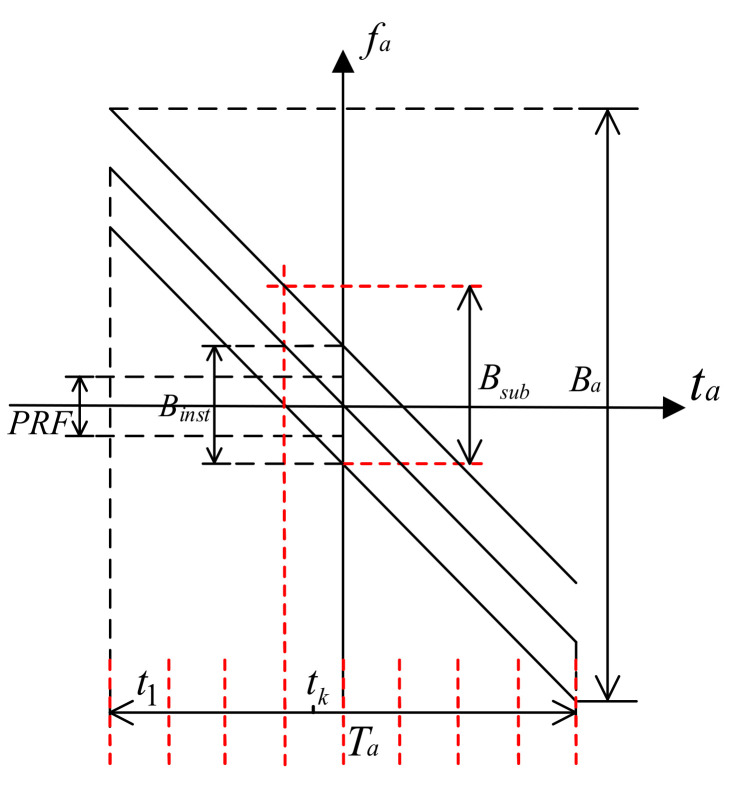
Time frequency diagram of a single equivalent phase center.

If there are *Q* satellites to receive data, the MIMO-SAR system can theoretically reduce PRF to 1/*Q* of the Doppler bandwidth. Figure 2 shows the time–frequency diagram of the data received by a single equivalent phase center. $B_{a}$ is the full aperture Doppler bandwidth of the scene, $B_{inst}$ is the instantaneous Doppler bandwidth, the edge real oblique line represents the time-frequency relationship of the edge point of the scene, and the middle real oblique line represents the time-frequency relationship of the scene center. The expression of $B_{a}$ is

(6) $$B_{a}\left(m\right)=-K_{a}\left(m\right)T_{a}+B_{inst}$$

where $K_{a}\left(m\right)=-2v^{2}f_{c}\left(m\right)/cR_{s}$ is Doppler rate. The first term in Equation (6) is the Doppler bandwidth caused by beam steering. The scene Doppler bandwidth of all equivalent phase centers is the union of the scene Doppler bandwidths of a single equivalent phase center, and can be expressed as

(7) $$B_{all}=-K_{a}\left(Q\right)T_{a}+B_{inst}$$

If the full aperture data are processed directly, PRF needs to be larger than $B_{all}/Q$. If the full aperture is divided into *K* segments, the Doppler bandwidth of *k*th sub-aperture signal is about [19]:

(8) $$B_{sub}\approx -\frac{K_{a}T_{a}}{K}+B_{inst}+\left(K_{a}(1)-K_{a}(Q)\right)t_{k}$$

where $K_{a}=-2v^{2}f_{c}/cR_{s}$ is the Doppler rate of the central satellite. Combined with MIMO-SAR theory, PRF only needs to conform to Equation (11) to remove the ambiguity of the sub-aperture signal.

(9) $$PRF\geq \frac{B_{sub}}{Q}$$

Therefore, PRF of sub aperture processing is $B_{all}/B_{sub}$ of full-aperture PRF, which greatly reduces the amount of echo data collected by satellite.

## 4. Signal Processing Flow

Since PRF in Equation (9) is less than $B_{sub}$, Doppler ambiguity will appear in sub-aperture signal. Before imaging, we need to reconstruct the sub-aperture signal without ambiguity and ultra-wide bandwidth. In this paper, first, the spatial filtering technique is used to remove the azimuth ambiguity of the sub-aperture signal, and then the improved TBS method is used to synthesize the bandwidth. Next, the CS-dechirp algorithm is used to process the sub-aperture reconstruction signal to obtain the sub-aperture low-resolution complex image. Finally, the sub-aperture complex image is fused coherently to obtain a high-resolution image corresponding to the full aperture. The following is a detailed theoretical derivation of each step.

### 4.1. Azimuth Deblurring Processing Based on Spatial Filtering

It can be seen from Figure 2 that the Doppler center of the signal in the *k*th sub aperture is $K_{a}t_{k}$. We first need to compensate the Doppler center to 0. The Doppler center compensation function can be constructed as

(10) $$H_{d}(t_{sub})=exp\left(-j2\pi f_{dc}\left(t_{sub}+t_{k}\right)\right)$$

where $f_{dc}=K_{a}t_{k}$ is the Doppler center. The later spatial filtering operation is similar to that of full aperture. The spatial filtering technology has been described in detail in references [15,16,17,18,19]. This paper only describes it briefly as a part of the imaging process. Taking the third order fuzzy, *Q* = 3 as an example, according to the spatial filtering principle, the following weight vector can be constructed.

(11) $$W\left(m\right)={\left[\begin{array}{ccc} w(m,1) & w(m,2) & w(m,3) \end{array}\right]}^{-1}$$

where

(12) $$w(m,n)={\left[\begin{array}{ccc} \exp\left(j2\pi \frac{f_{a}+f_{dc}-PRF}{v}x_{m,n}\right) & \exp\left(j2\pi \frac{f_{a}+f_{dc}}{v}x_{m,n}\right) & \exp\left(j2\pi \frac{f_{a}+f_{dc}+PRF}{v}x_{m,n}\right) \end{array}\right]}^{T}$$

After the signal is transformed into Doppler domain, the azimuth unambiguous signal can be recovered by filtering the same sub-band signal with the weight vector.

(13) $$[S_{m}\left(f_{a}-PRF\right),S_{m}\left(f_{a}\right),S_{m}\left(f_{a}+PRF\right)]=[S_{m,1}\left(f_{a}\right),S_{m,2}\left(f_{a}\right),S_{m,3}\left(t,f_{a}\right)]W\left(m\right)$$

where $S_{m}\left(f_{a}\right)$ is the unambiguous signal, which can be expressed as

(14) $$\begin{array}{ll} S_{m}\left(f_{a}\right)= & a_{r}\left(\hat{t}-\frac{2R\left(f_{a}+f_{dc}\right)}{c}\right)\exp\left(j\pi \gamma_{e}{\left(\hat{t}-\frac{2R\left(f_{a}+f_{dc}\right)}{c}\right)}^{2}\right)a_{a}\left(f_{a}+f_{dc}\right)\\ & \times \exp\left(-\frac{2\pi }{v}R_{B}\sqrt{f_{aM}{\left(m\right)}^{2}-{\left(f_{dc}+f_{a}\right)}^{2}}-2\pi \left(f_{dc}+f_{a}\right)\frac{X}{v}+2\pi f_{a}t_{k}\right) \end{array}$$

where $f_{aM}\left(m\right)=2vf_{c}\left(m\right)/c$. After obtaining the unambiguous azimuth signal, it is necessary to synthesize sub-band signals into a large bandwidth signal in range. In this paper, the improved TBS method is used to realize the bandwidth synthesis.

### 4.2. Improved TBS Method

The traditional TBS method has four steps: frequency shift, phase correction, time shift, and superposition of frequency bands. Its process is complicated and the calculation is complex. The traditional TBS does not eliminate the quadratic phase of range frequency before the frequency shift. Therefore, the quadratic term will also shift after the frequency shift, and the offset quadratic term will fail to accumulate the spectrum coherently. Therefore, the traditional TBS needs phase correction and time shift to eliminate the negative effect of the quadratic phase offset. If the quadratic phase of frequency in range is eliminated before frequency shift, the steps of bandwidth synthesis will be greatly simplified. Paper [19] improves the FBS algorithm on the basis of eliminating the quadratic phase. In [19], the range compression is first carried out, and then the frequency shift processing is divided into two steps: the slight shift of fractional frequency-bin interval in time domain and the frequency shift of an integer number of frequency bins in the frequency domain.

Inspired by paper [19], this paper also eliminates the quadratic phase of the range frequency before the frequency shift. The two-step processing of frequency shift in [19] is reduced to one-step processing in time domain in this paper, so as to get an improved TBS method. The improved TBS first transforms the deblurred signal into dual-frequency domain, and then compensates the range-frequency quadratic phase. The signal becomes

(15) $$\begin{array}{ll} S_{m}\left(f_{r},f_{a},t_{k}\right)= & a_{r}\left(\frac{f_{r}}{B}\right)a_{a}\left(f_{a}+f_{dc}\right)\exp\left(j2\pi f_{a}t_{k}\right)\\ & \times \exp\left(-j4\pi R_{B}\sqrt{\left({\left(\frac{f_{c}\left(m\right)+f_{r}}{c}\right)}^{2}-{\left(\frac{f_{a}}{2v}\right)}^{2}\right)}-\frac{2\pi \left(f_{a}+f_{dc}\right)}{v}X\right) \end{array}$$

Then, IFFT in range is made for Equation (15), and the signal becomes

(16) $$\begin{array}{ll} S_{m}\left(\hat{t},f_{a},t_{k}\right)= & \delta_{r}\left(\hat{t}-2R\left(f_{a}+f_{dc}\right)/c\right)a_{a}\left(f_{a}+f_{dc}\right)\exp\left(j2\pi f_{a}t_{k}\right)\\ & \times \exp\left(-j4\pi R_{B}\sqrt{{\left(\frac{f_{c}\left(m\right)}{c}\right)}^{2}-{\left(\frac{f_{a}+f_{dc}}{2v}\right)}^{2}}-\frac{2\pi \left(f_{a}+f_{dc}\right)}{v}X\right) \end{array}$$

Next, the frequency shift is carried out in the range time domain. The frequency shift function can be constructed as

(17) $$H_{shift}\left(\hat{t},m\right)=\exp\left(j2\pi \left(m-\frac{1}{2}-\frac{Q}{2}\right)B\hat{t}\right),\;m=1,\ldots ,Q$$

Equations (16) and (17) are multiplied to complete the frequency shift. After that, a range FFT is performed on the signal, and then the signal becomes

(18) $$\begin{array}{ll} S_{m}\left(f_{r},f_{a}\right)= & a_{r}\left(\left(f_{r}-\left(m-\left(1+Q\right)/2\right)B\right)/B\right)a_{a}\left(f_{a}+f_{dc}\right)\exp\left(j2\pi f_{a}t_{k}\right)\\ & \times \exp\left(-j4\pi R_{B}\sqrt{{\left(\frac{f_{c}+f_{r}}{c}\right)}^{2}-{\left(\frac{f_{a}+f_{dc}}{2v}\right)}^{2}}-\frac{2\pi \left(f_{a}+f_{dc}\right)}{v}X\right) \end{array}$$

After the above processing, the signal with whole bandwidth can be obtained by coherently accumulating all sub-band signals. The signal with whole bandwidth can be expressed as

(19) $$\begin{array}{ll} S\left(f_{r},f_{a},t_{k}\right) & =\sum_{m=1}^{Q}S_{m}\left(f_{r},f_{a}\right)\\ & =a_{r}\left(\frac{f_{r}}{QB}\right)a_{a}\left(f_{a}+f_{dc}\right)\exp\left(j2\pi f_{a}t_{k}\right)\\ & \times \exp\left(-j4\pi R_{B}\sqrt{{\left(\frac{f_{c}+f_{r}}{c}\right)}^{2}-{\left(\frac{f_{a}+f_{dc}}{2v}\right)}^{2}}-\frac{2\pi \left(f_{a}+f_{dc}\right)}{v}X\right) \end{array}$$

It can be seen from Equation (19) that after the bandwidth synthesis, the bandwidth in range is expanded from B to QB, and unambiguous and full bandwidth sub-aperture signal is reconstructed. Then the imaging algorithm based on sub-aperture image fusion is used to image the reconstructed signal.

**Figure 3:**
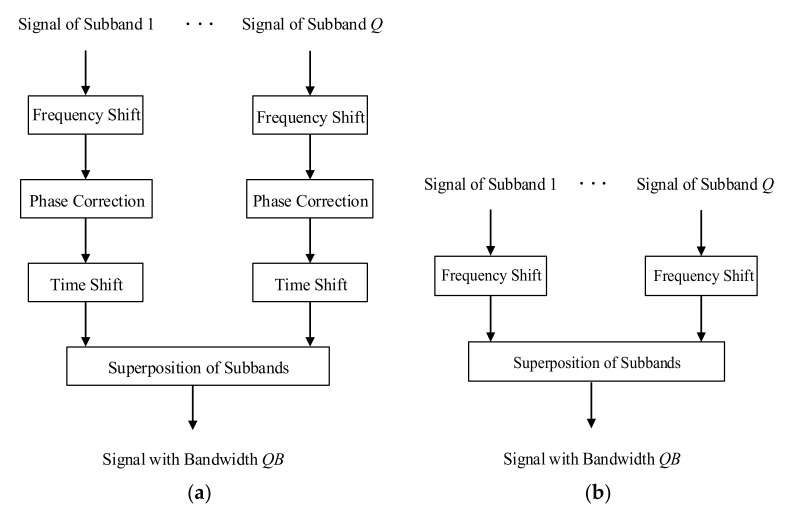
Comparison of two time domain bandwidth synthesis (TBS) methods. (**a**) Traditional TBS; (**b**) improved TBS.

Figure 3 shows the flow chart of two TBS methods. As shown in Figure 3, the improved TBS method eliminates the phase correction and time shift of the traditional TBS method, which makes data processing more efficient. Suppose $N_{r}$ and $N_{a}$ denote the range and azimuth sampling numbers of each sub-band signal, respectively, according to the flow of TBS method, the computational load of improved TBS method can be written as $\left(1/2\right)QN_{r}N_{a}\log_{2}N_{r}+QN_{r}N_{a}$, while the computational load of conventional TBS method is $\left(1/2\right)QN_{r}N_{a}\log_{2}N_{r}+3QN_{r}N_{a}$. The improved TBS reduces the computational load of $2QN_{r}N_{a}$. When $N_{r}$ and $N_{a}$ are large (in the ultra-high resolution mode), the reduction of calculation is huge.

### 4.3. Imaging Algorithm Based on Sub-Aperture Image Fusion

Reference [29] proposes an imaging method based on sub-aperture image fusion for strip monostatic SAR. We apply the method to spotlight MIMO-SAR. First, the CS algorithm is used for range cell migration correction and range compression for the reconstructed sub-aperture signal. Then, dechirp operation is performed in azimuth to obtain the complex sub-aperture image with low resolution. Finally, all sub-aperture images are fused coherently to obtain azimuth full-resolution image.

#### 4.3.1. Two-Dimensional Focusing Processing Based on CS-Dechirp

Due to the limited space, this paper directly presents the result of range cell migration correction and range compression using CS algorithm. The specific process can be seen in [29]. The signal after migration compensation and range compression can be expressed as

(20) $$\begin{array}{ll} S(\hat{t},f_{a},t_{k}) & =\delta_{r}(t-\frac{2R_{B}}{c})a_{a}(f_{a}+f_{dc})\\ & \times \exp\left(-j\frac{2\pi }{v}R_{B}\sqrt{f_{aM}{}^{2}-{\left(f_{a}+f_{dc}\right)}^{2}}\right)exp\left(-j2\pi (f_{a}+f_{dc})\frac{X}{v}\right) \end{array}$$

Construction of compensation function $H_{1}$ for hyperbolic phase transition

(21) $$H_{1}(f_{a},t_{k})=\exp\left(j\frac{2\pi }{v}R_{B}\sqrt{f_{aM}{}^{2}-{\left(f_{a}+f_{dc}\right)}^{2}}-j\frac{\pi }{K_{a}}{\left(f_{a}+f_{dc}\right)}^{2}\right)$$

By multiplying Equation (20) with $H_{1}$, the hyperbolic phase of the signal is transformed into a quadratic phase. Then an azimuth IFFT is performed on the signal, and the signal becomes

(22) $$\begin{array}{ll} S(\hat{t},t_{sub},t_{k}) & =\delta_{r}(\hat{t}-\frac{2R_{B}}{c})a_{a}(t_{sub})\\ & \times \exp\left(j\pi K_{a}{\left(t_{sub}+t_{k}-\frac{X}{v}\right)}^{2}-j2\pi f_{dc}\left(t_{sub}+t_{k}\right)\right) \end{array}$$

Then the dechirp function $H_{2}$ is constructed. The dechirp operation can be completed by multiplying Equation (22) with *H*_2_.

(23) $$H_{2}(t_{sub},t_{k})=\exp\left(-j\pi K_{a}{\left(t_{sub}+t_{k}\right)}^{2}\right)$$

Since the Doppler center of Equation (22) is 0 and the Doppler center of $H_{2}$ is $f_{dc}{}^{’}=-K_{a}\times t_{k}$, the phase center of the signal will change to $f_{dc}{}^{’}$ after dechirp. At this time, azimuth FFT will cause Doppler overlap. Therefore, it is necessary to compensate the Doppler center of the signal after dechirp to 0. Combined with $f_{dc}=K_{a}t_{k}$, the compensation function of Doppler center is

(24) $$H_{3}(t_{sub},t_{k})=\exp\left(j2\pi f_{dc}\left(t_{sub}+t_{k}\right)\right)$$

After multiplying Equation (22) by $H_{2}$ and $H_{3}$, the low-resolution complex image focused in $\hat{t}-f_{a}$ domain can be obtained by azimuth FFT as

(25) $$\begin{array}{ll} S(\hat{t},f_{a},t_{k}) & =\delta_{r}(\hat{t}-\frac{2R_{B}}{c})\delta_{a}\left(f_{a}+K_{a}\frac{X}{v}\right)\\ & \times \exp\left(-j2\pi K_{a}\frac{X}{v}t_{k}\right)\exp\left(j\pi K_{a}{\left(\frac{X}{v}\right)}^{2}\right) \end{array}$$

#### 4.3.2. Coherent Fusion of Sub-Aperture Complex Images

According to Equation (25), the phase of the sub-aperture focusing signal is linear with respect to $t_{k}$. However, the linear phase in Equation (25) is not a constant at the focusing frequency point $f_{a0}=-K_{a}X/v$, which leads to the different phases of the focusing signals of different sub apertures at $f_{a0}$, resulting in the coherent accumulation of sub aperture images. As a result, the sub-aperture images cannot be coherently accumulated. Therefore, a phase compensation function $H_{4}$ can be constructed as

(26) $$H_{4}(f_{a},t_{k})=\exp\left(-j2\pi f_{a}t_{k}\right)$$

After multiplying Equation (25) with $H_{4}$, the signal becomes

(27) $$\begin{array}{ll} S(\hat{t},f_{a},t_{k}) & =\delta_{r}(\hat{t}-\frac{2R_{B}}{c})\delta_{a}\left(f_{a}+K_{a}\frac{X}{v}\right)\\ & \times \exp\left(-j2\pi \left(f_{a}+K_{a}\frac{X}{v}\right)t_{k}\right)\exp\left(j\pi K_{a}{\left(\frac{X}{v}\right)}^{2}\right) \end{array}$$

It can be seen from Equation (27) that the phase of each sub-aperture focusing signal is 0 at $f_{a0}$ and the phase at the non-focusing point is linear with $t_{k}$. The phase of each sub-aperture focusing signal is coherent with that of other sub-aperture focusing signals. Thus, the accumulation of sub-aperture signals is coherent and the amplitude of the focus is enhanced. Accordingly, the resolution is improved.

**Figure 4:**
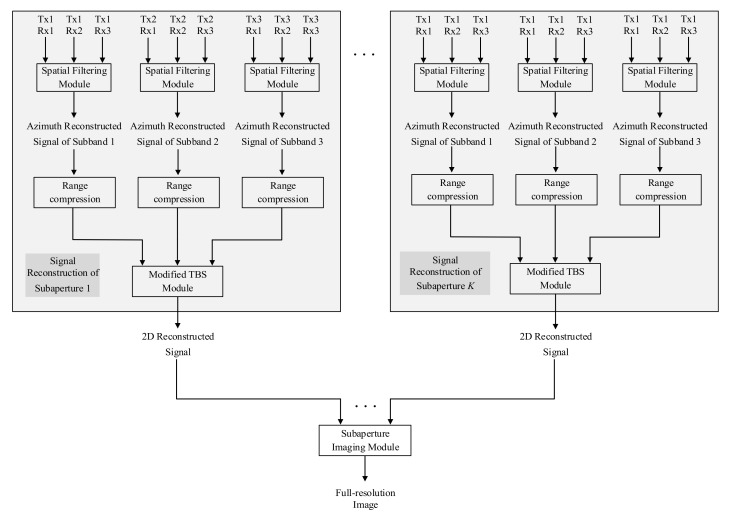
Signal processing flow chart of the model of three transmitters and three receivers.

In conclusion, the signal processing flow chart of the ultra-high resolution imaging method is shown in Figure 4. TxmRxn in the figure represents the signal transmitted by satellite *m* and received by satellite *n*.

## 5. Simulation Experiment and Result Analysis

In this section, the imaging simulation on point targets and distributed targets is carried out to verify the effectiveness of the proposed method. The bandwidth synthesis experiment is performed to verify the effectiveness of the improved TBS method. The main parameters of the simulation are shown in Table 1. The parameters in Table 1 are designed based on known reference data [31,32].

### 5.1. Bandwidth Synthesis Experiment

**Figure 5:**
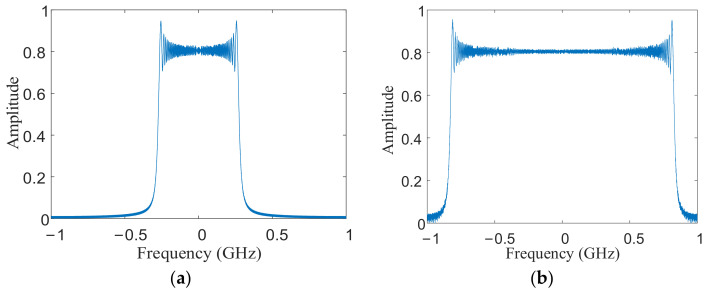
Bandwidth synthesis of single point target. (**a**) sub-band bandwidth; (**b**) synthesized bandwidth.

This section uses the improved TBS method to complete the bandwidth synthesis to verify the effectiveness of the improved TBS method. Because it only needs to show the result of bandwidth synthesis, the simulation in this section adopts the multi-transmitter and single-receiver model, and there is no design of azimuth ambiguity (the value of PRF is three times of that in Table 1). Three satellites transmit signals with different carrier frequencies. The step frequency between the carrier frequencies is shown in Table 1. The central satellite serves as a receiving satellite to receive signals from three satellites. In order to show the range frequency spectrum better, a single point target is used for bandwidth synthesis experiment. the point target is located in the center of the scene. After receiving all the transmitted signals, we use band-pass filter to separate the echo signal and get 3 sub-band signals. Then, the improved TBS method is used to synthesize the sub-band signals into a signal with large bandwidth. The synthesis steps are described in detail in Section 4.2. Figure 5 shows the result of bandwidth synthesis for point target. As shown in Figure 5, the sub-band signals are well combined into a large-bandwidth signal by the improved TBS method, and the synthesized bandwidth is approximately three times of the sub-band bandwidth. This proves that synthesis effect of the improved TBS method is excellent.

The improved TBS method simplifies the processing flow of traditional TBS method. In order to illustrate the benefits of this simplification, we use two TBS methods to carry out the experiments of bandwidth synthesis experiments in MATLAB, and compare the running time of the two methods. In order to show the difference in the running time of the two TBS methods under different data volume, we set up four experiments, and sampling points of the four experiments are different (in order to obtain different sampling points, the signal bandwidth and synthetic aperture time of the four experiments are different). Table 2 shows the average running time of four experiments in MATLAB. It can be seen from Table 2 that compared with the traditional TBS method, the improved TBS method can reduce the execution time of bandwidth synthesis. The reduction of time is about 30%~40%. Compared with the traditional TBS method, the running time of the improved TBS method is reduced by 30%–40%. When the amount of data is huge, the time saved is very much.

### 5.2. Simulation on Point Targets

**Figure 6:**
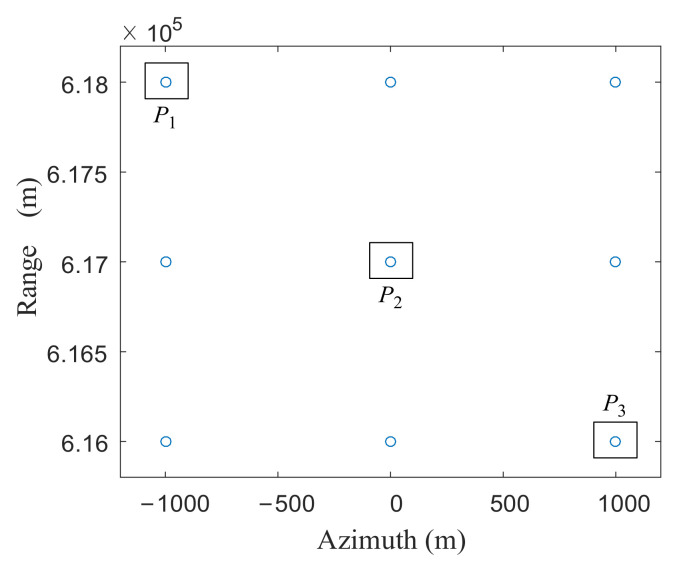
Lattice distribution of the point targets.

After the effectiveness of the improved TBS method is verified, the imaging simulation of the lattice targets is carried out. The system model used in the simulation is shown in Figure 1 in Section 2. Each satellite transmits LFM signals with different carrier frequencies, and receives signals from all three satellites. Figure 6 shows the distribution of the lattice: there are 3×3 evenly distributed lattice on the ground, and the distance and azimuth point spacing are 1 km. The coordinates of the three points, marked in Figure 6, are $P_{1}\left(-1\;\mathrm{km},R_{s}-1\;\mathrm{km}\right)$, $P_{2}\left(0,R_{s}\right)$ and $P_{3}\left(1\;\mathrm{km},R_{s}+1\;\mathrm{km}\right)$. $R_{s}$ is the vertical slant range of the scene center.

**Figure 7:**
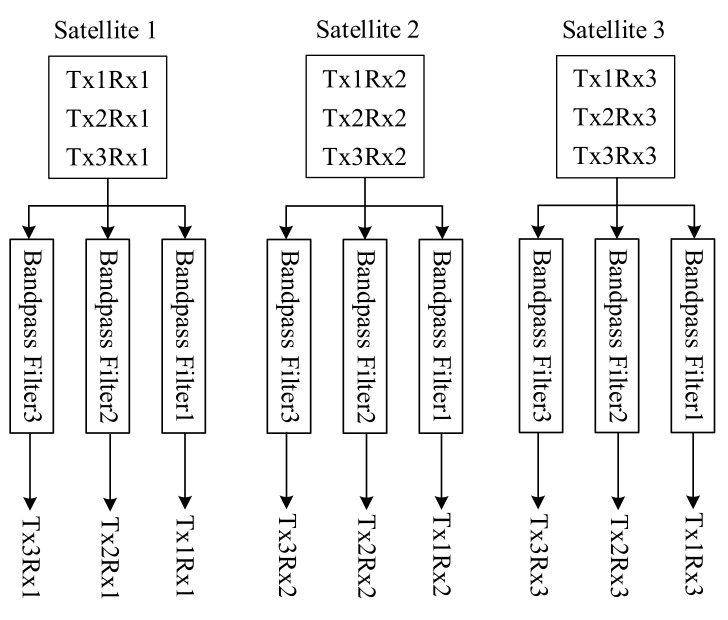
Signal separation

After all the signals are received by three receiving satellites, the received signal of each satellite is separated into three sub-band signals by band-pass filter. Three sub-band signals can be separated from each satellite. Then nine sub-band signals can be separated from the three satellites. The schematic diagram of signal separation is shown in Figure 7. TxmRxn is the signal transmitted by satellite *m* and received by satellite *n*.

**Figure 8:**
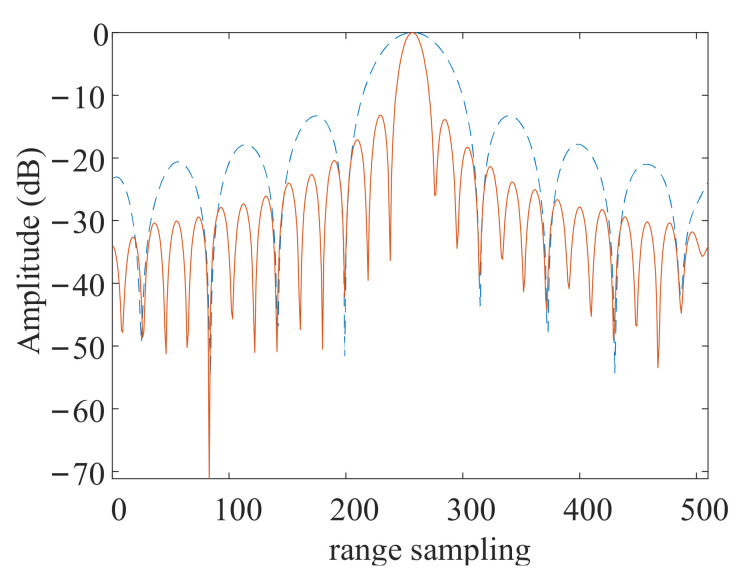
Range profile before and after frequency band synthesis of point target $P_{1}$.

After the sub-band signal is separated, the signal is processed according to the process shown in Figure 4. The spatial filter is constructed according to Equation (11). Among the nine output signals shown in Figure 7, every three output signals are in the same sub band. The signals in the same sub band are used as input of the spatial filter to get unambiguous sub-band signal. So there are three sets of inputs of spatial filter, and we can get three unambiguous signals which are in different sub bands. Three unambiguous sub-band signals are used as the inputs of the improved TBS module to obtain a large bandwidth signal. Then the unambiguous signal with large bandwidth is used as the input of sub-aperture imaging module. In the sub-aperture imaging module, the signal is processed by CS-dechirp to get the sub-aperture image with low azimuth resolution (the detailed processing flow is described in Section 4.3.1). Figure 8 shows the range profile of a point target in sub-aperture image. The red solid line in Figure 8 is the distance profile obtained after the above steps. The blue dotted line in Figure 8 is the result without bandwidth synthesis when the unambiguous sub-band signal is directly used as the input of the sub-aperture imaging module. From the comparison between the dotted line and the solid line in Figure 8, we can find that the range resolution is improved after bandwidth synthesis, which further verifies the effectiveness of the improved TBS method.

**Figure 9:**
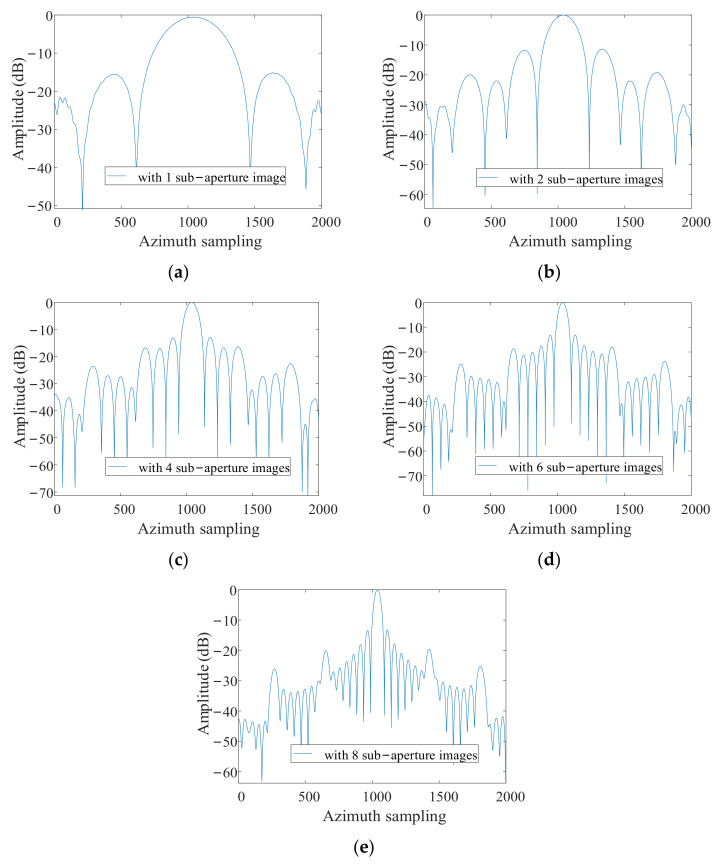
Azimuth profile of point target $P_{1}$ (**a**) with 1 sub-aperture image; (**b**) with 2 sub-aperture images; (**c**) with 4 sub-aperture images; (**d**) with 6 sub-aperture images; (**e**) with 8 sub-aperture images.

**Figure 10:**
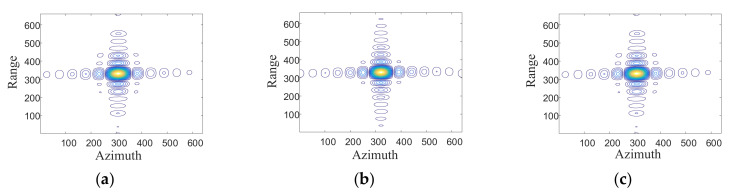
Contour plots of three of point targets. (**a**) contour plot of $P_{1}$ (**b**) contour plot of $P_{2}$; (**c**) contour plot of $P_{3}$.

After obtaining the sub-aperture image, we change the phase of the image to coherent (the processing method has been described in Section 4.3.2). Then all sub-aperture images are fused coherently. The way of fusion is to map sub images to a grid image, in which the interval between the azimuth sampling points equals to the resolution corresponding to the full-aperture. Figure 9 shows the change of the azimuth resolution of the grid image during the fusion process. After 1, 2, 4, 6, and 8 sub-aperture images are fused, the resolution of the grid image is presented in Figure 9a–e respectively (In this simulation, the full aperture is divided into 8 sub apertures). As can be seen from Figure 9a–e, the azimuth resolution of the grid image becomes higher and higher with the sub-aperture data stream coming. The two-dimensional imaging result of point target is shown in Figure 10.

Figure 10 shows the contour map of three point targets $P_{1}$, $P_{2}$, and $P_{3}$ in the grid image after fusion. The peak side-lobe ratio (PSLR) and integral side-lobe ratio (ISLR) of the imaging results of these point targets are presented in Table 3. It can be seen from Figure 10 and Table 3 that both the center point ($P_{2}$) and the edge points ($P_{1}$ and $P_{3}$) of the scene can be imaged well, which indicates that the imaging performance of the proposed method is good.

**Figure 11:**
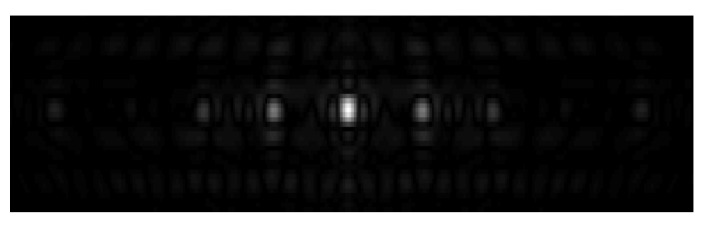
Simulation result of point target of the full-aperture multiple input multiple output-synthetic aperture radar (MIMO-SAR) algorithm.

Figure 11 shows the simulation result of the point target of the full-aperture MIMO-SAR algorithm [15,16,17] under the parameters shown in Table 1. In Figure 11, there are multiple phantoms in the azimuth. This is because the PRF designed in this simulation is very small (compared with the Doppler bandwidth), and the ambiguity times of the full-aperture signal are more than three times. Thus, three satellites cannot get enough spatial degree of freedom to eliminate azimuth ambiguity. If the full-aperture MIMO-SAR algorithm can image correctly, the PRF cannot be less than 1/3 of the full-aperture Doppler bandwidth. APC algorithm [12,13,14] is similar. Its PRF is larger than Doppler bandwidth, so it cannot image correctly under the parameters shown in Table 1, as well.

So the cost for correct imaging of the full-aperture MIMO-SAR algorithm and APC algorithm is to increase the PRF, which will obviously increase the amount of data collected by the satellite. Taking the parameters in Table 1 as an example, the full-aperture Doppler bandwidth of the point target is 73,897 Hz (calculated by $-K_{a}\left(m\right)T_{a}$, which has been explained in the Equation (6)). Full-aperture MIMO-SAR algorithm requires PRF>24,632 Hz. APC algorithm requires PRF>73,897 Hz. Table 4 lists the number of azimuth sampling points and PRF required by full-aperture MIMO-SAR algorithm, APC algorithm, and the proposed algorithm for correct imaging. In Table 4, $N_{a}$ represents the number of azimuth sampling points ($N_{a}=PRF\times T_{a}$). PRF of the full-aperture MIMO-SAR algorithm and APC algorithm is 1.2 times of the minimum requirement. PRF of the proposed algorithm is determined according to Table 1.

It can be seen from Table 4 that reduction in the number of azimuth sampling points of the proposed method is huge. Thus, the amount of data collected by satellite is greatly reduced, which is the advantage of the proposed method.

### 5.3. Simulation on Distributed Targets

**Figure 12:**
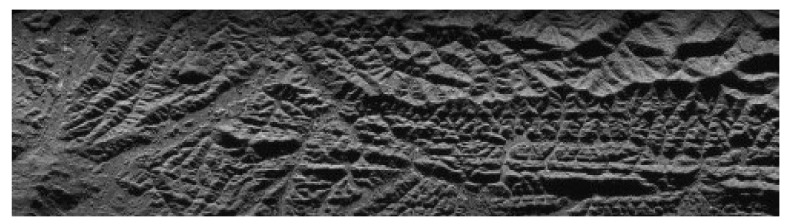
SAR image for echo simulation.

Since there are no spaceborne MIMO-SAR data to use, this paper takes a SAR image as distributed target for the echo simulation. The pixels of the image are arranged in the slant plane as point targets. The main parameters of the simulation are shown in Table 1. The system model used in the simulation is shown in Figure 1. The simulation process is the same as that of point targets. The SAR image as distributed targets in the simulation is shown in Figure 12.

**Figure 13:**
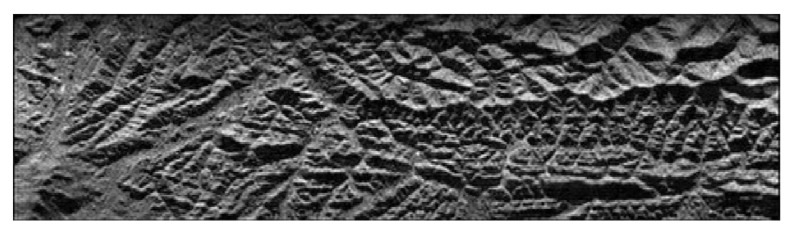
Imaging result of distributed target.

After processing the echo according to the algorithm proposed in this paper, the imaging result is shown in Figure 13. The imaging result is basically consistent with the original image, which verifies the effectiveness of the proposed algorithm.

## 6. Conclusions

An ultra-high resolution imaging method for distributed small satellite spotlight MIMO-SAR has been proposed in this paper. Moreover, an improved TBS method is put forward for bandwidth synthesis. The algorithm directly reconstructs and images the sub-aperture signal. Moreover, PRF only needs to be able to recover the sub-aperture unambiguous signal, which greatly reduces the risk of ambiguity in range and the amount of echo data. In addition, an improved TBS method is provided for bandwidth synthesis, which simplifies the operation process of traditional TBS and improves the efficiency of frequency band synthesis. Simulation results show the feasibility and effectiveness of this method.

## Figures and Tables

**Table 1:** Simulation parameters.

Parameters	Value
Number of satellites	3
Platform velocity	7391 m/s
Center line distance	617 km
Step frequency	500 MHz
PRF	5000 Hz
Signal bandwidth	500 MHz
Synthetic aperture time	13.04 s
Number of sub apertures	8
Center frequency of whole bandwidth	9.6 GHz
Resolution	0.1 m
Baseline distance	500 m

**Table 2:** Running time of TBS.

Range Sampling Numbers	Azimuth Sampling Numbers	Average Running Time of 5 TBS Experiments (s)	Average Running Time of 5 Improved TBS Experiments (s)
8192	65,536	17,961	11,164
4096	32,768	10,705	7203
4096	16,384	531	356
4096	8192	342	221

**Table 3:** Peak side-lobe ratio (PSLR) and integral side-lobe ratio (ISLR) of point targets.

Point Target	Range		Azimuth	
	PSLR (dB)	ISLR (dB)	PSLR (dB)	ISLR (dB)
*P*_1_	−13.60	−10.35	−13.23	−9.87
*P*_2_	−13.61	−10.37	−13.23	−9.77
*P*_3_	−13.33	−9.75	−13.88	−10.71

**Table 4:** Number of azimuth sampling points and pulse repetition rate (PRF) of different algorithms.

Algorithm	PRF (Hz)	Na
Full-aperture MIMO-SAR Algorithm	89,000	1,160,560
APC Algorithm	30,000	391,200
Proposed Algorithm	5000	65,200

## Data Availability

Not applicable.
